# Autoimmune Encephalitis Misdiagnosis in Adults

**DOI:** 10.1001/jamaneurol.2022.4251

**Published:** 2022-11-28

**Authors:** Eoin P. Flanagan, Michael D. Geschwind, A. Sebastian Lopez-Chiriboga, Kyle M. Blackburn, Sanchit Turaga, Sophie Binks, Jennifer Zitser, Jeffrey M. Gelfand, Gregory S. Day, S. Richard Dunham, Stefanie J. Rodenbeck, Stacey L. Clardy, Andrew J. Solomon, Sean J. Pittock, Andrew McKeon, Divyanshu Dubey, Anastasia Zekeridou, Michel Toledano, Lindsey E. Turner, Steven Vernino, Sarosh R. Irani

**Affiliations:** 1 Laboratory Medicine and Pathology, Mayo Clinic College of Medicine, Rochester, Minnesota; 2 Center for Multiple Sclerosis and Autoimmune Neurology, Department of Neurology, Mayo Clinic College of Medicine, Rochester, Minnesota; 3 Department of Neurology, University of California, San Francisco (UCSF), San Francisco; 4 Department of Neurology, Mayo Clinic, Jacksonville, Florida; 5 Department of Neurology, University of Texas Southwestern Medical Center, Dallas; 6 Autoimmune Neurology Group, West Wing, Level 3, John Radcliffe Hospital, University of Oxford, Oxford, United Kingdom; 7 Movement Disorders Unit, Department of Neurology, Tel Aviv Sourazky Medical Center, Affiliate of Sackler Faculty of Medicine, Tel-Aviv University, Tel-Aviv, Israel; 8 Washington University in St Louis, St Louis, Missouri; 9 Department of Neurology, University of Utah, Salt Lake City; 10 Larner College of Medicine at the University of Vermont, Burlington; 11 Graduate School of Health Sciences, Mayo Clinic College of Medicine, Rochester, Minnesota

## Abstract

**Importance:**

Autoimmune encephalitis misdiagnosis can lead to harm.

**Objective:**

To determine the diseases misdiagnosed as autoimmune encephalitis and potential reasons for misdiagnosis.

**Design, Setting, and Participants:**

This retrospective multicenter study took place from January 1, 2014, to December 31, 2020, at autoimmune encephalitis subspecialty outpatient clinics including Mayo Clinic (n = 44), University of Oxford (n = 18), University of Texas Southwestern (n = 18), University of California, San Francisco (n = 17), University of Washington in St Louis (n = 6), and University of Utah (n = 4). Inclusion criteria were adults (age ≥18 years) with a prior autoimmune encephalitis diagnosis at a participating center or other medical facility and a subsequent alternative diagnosis at a participating center. A total of 393 patients were referred with an autoimmune encephalitis diagnosis, and of those, 286 patients with true autoimmune encephalitis were excluded.

**Main Outcomes and Measures:**

Data were collected on clinical features, investigations, fulfillment of autoimmune encephalitis criteria, alternative diagnoses, potential contributors to misdiagnosis, and immunotherapy adverse reactions.

**Results:**

A total of 107 patients were misdiagnosed with autoimmune encephalitis, and 77 (72%) did not fulfill diagnostic criteria for autoimmune encephalitis. The median (IQR) age was 48 (35.5-60.5) years and 65 (61%) were female. Correct diagnoses included functional neurologic disorder (27 [25%]), neurodegenerative disease (22 [20.5%]), primary psychiatric disease (19 [18%]), cognitive deficits from comorbidities (11 [10%]), cerebral neoplasm (10 [9.5%]), and other (18 [17%]). Onset was acute/subacute in 56 (52%) or insidious (>3 months) in 51 (48%). Magnetic resonance imaging of the brain was suggestive of encephalitis in 19 of 104 patients (18%) and cerebrospinal fluid (CSF) pleocytosis occurred in 16 of 84 patients (19%). Thyroid peroxidase antibodies were elevated in 24 of 62 patients (39%). Positive neural autoantibodies were more frequent in serum than CSF (48 of 105 [46%] vs 7 of 91 [8%]) and included 1 or more of GAD65 (n = 14), voltage-gated potassium channel complex (LGI1 and CASPR2 negative) (n = 10), *N*-methyl-d-aspartate receptor by cell-based assay only (n = 10; 6 negative in CSF), and other (n = 18). Adverse reactions from immunotherapies occurred in 17 of 84 patients (20%). Potential contributors to misdiagnosis included overinterpretation of positive serum antibodies (53 [50%]), misinterpretation of functional/psychiatric, or nonspecific cognitive dysfunction as encephalopathy (41 [38%]).

**Conclusions and Relevance:**

When evaluating for autoimmune encephalitis, a broad differential diagnosis should be considered and misdiagnosis occurs in many settings including at specialized centers. In this study, red flags suggesting alternative diagnoses included an insidious onset, positive nonspecific serum antibody, and failure to fulfill autoimmune encephalitis diagnostic criteria. Autoimmune encephalitis misdiagnosis leads to morbidity from unnecessary immunotherapies and delayed treatment of the correct diagnosis.

## Key Points

**Question:**

What diseases are misdiagnosed as autoimmune encephalitis and which factors contribute to misdiagnosis?

**Findings:**

In this case series of 107 outpatients misdiagnosed with autoimmune encephalitis, approximately half had functional neurologic or psychiatric disorders. An insidious rather than subacute onset and lack of magnetic resonance imaging or cerebrospinal fluid findings suggestive of inflammation were clues to misdiagnosis; overinterpretation of serum nonspecific antibodies was a major contributor to misdiagnosis.

**Meaning:**

A broad range of disorders are misdiagnosed as autoimmune encephalitis and misdiagnosis occurs in many settings including at specialized centers participating in this study.

## Teaser

This case series assesses the diseases misdiagnosed as autoimmune encephalitis and potential reasons for misdiagnosis.

## Introduction

Autoimmune encephalitis is increasingly a diagnostic consideration in patients with subacute onset of memory loss, altered mental status, and/or psychiatric symptoms—core features of proposed diagnostic criteria.^1^ Detection of autoimmune encephalitis is increasing over time with new neural autoantibody biomarker discovery and greater awareness among clinicians, although the diagnosis remains rare overall.^2^ Diagnostic mimics of autoimmune encephalitis are far more prevalent than autoimmune encephalitis, including toxic/metabolic encephalopathies, functional neurological disorders, primary psychiatric disease, neurodegenerative disorders, neoplasms, and epilepsy.^2,3^ Although discovery of novel antineuronal and antiglial autoantibodies has improved diagnostic sensitivity for autoimmune encephalitis, specificity varies by antibody type, test methodology, and pretest probability.^4^ Thus, there is a potential for false-positive autoantibody results in patients with diseases other than autoimmune encephalitis, which can contribute to misdiagnosis.^5,6,7^ In much of the autoimmune encephalitis literature, there is emphasis on patients in whom the diagnosis of autoimmune encephalitis was initially erroneously overlooked. Yet, there are limited data concerning patients initially incorrectly diagnosed with autoimmune encephalitis and their subsequent correct diagnosis. This is an important topic given the risk of patient harm associated with misdiagnosis, including morbidity from adverse effects of immunotherapies and delay of appropriate treatment.^8^ We report data from an international multicenter study of autoimmune encephalitis misdiagnosis across 6 subspecialty centers to analyze patients misdiagnosed with autoimmune encephalitis and identify possible contributors to misdiagnosis.

## Methods

The Mayo Clinic institutional review board approved this multicenter study (#19-004926), and institutional review board approval also occurred at each respective site with all patients either providing written consent or patients included under an institutional review board approved consent waiver for minimal risk retrospective studies. This study was a retrospective multicenter observational study that followed the Strengthening the Reporting of Observational Studies in Epidemiology (STROBE) reporting guideline for reporting observational studies.

### Inclusion Criteria

Inclusion criteria were adult patients (18 years or older) at the time of neurologic evaluation at a participating site with (1) a prior autoimmune encephalitis diagnosis assigned at another medical center or at the participating site and occurring in the inpatient or outpatient setting and (2) a subsequent alternative diagnosis made at an in-person visit at one of the participating outpatient autoimmune neurology clinics. Alternative diagnoses were defined as a definite alternative diagnosis when diagnostic testing confirmed the diagnosis (eg, brain biopsy revealing tumor) or as a clinical alternative diagnosis when definitive confirmation (eg, biopsy) was not available or it was a purely clinical diagnosis (eg, primary psychiatric disease).

### Patient Identification at Participating Centers and Frequency of Misdiagnosis vs Actual Autoimmune Encephalitis Diagnosis

Six academic medical centers with subspecialty expertise in autoimmune neurology participated. These included Mayo Clinic in Rochester, Minnesota (autoimmune neurology clinic); University of Oxford in Oxford, United Kingdom (autoimmune neurology clinic); University of Texas Southwestern in Dallas (autoimmune neurology clinic); University of California, San Francisco in San Francisco (Department of Neurology Multiple Sclerosis/Neuroinflammation clinic, the Memory and Aging Center clinic or through the Memory and Aging Center rapidly progressive dementia research program); Washington University in St Louis in St Louis, Missouri (rapidly progressive dementia/autoimmune encephalitis clinic); and University of Utah in Salt Lake City (autoimmune neurology clinic). Patients evaluated clinically between January 1, 2014, to December 31, 2020, were considered for study enrollment. Data on 2 patients included in the study were previously published in case reports.^9,10^ At the University of California San Fransisco, only patients who received immunotherapy for their presumed autoimmune encephalitis diagnosis were included. Details on numbers of true autoimmune encephalitis over the same study time frame, when available, were also collected to assess its frequency.

### Data Collection

Participating centers provided deidentified data detailing age, sex, clinical, and paraclinical variables from patients misdiagnosed with autoimmune encephalitis. Data on race and ethnicity were not collected. Data on the requirements for part 1 and part 2 of the diagnostic criteria for possible autoimmune encephalitis (a requirement for diagnosis of any autoimmune encephalitis category) were also specifically collected and include^1^ (1) subacute onset (rapid progression of <3 months) of working memory deficits (short-term memory loss), altered mental status, or psychiatric symptoms and (2) at least one of the following: new focal central nervous system findings, seizures not explained by a previously known seizure disorder, cerebrospinal fluid (CSF) pleocytosis (white blood cell count of >5 cells/mm^3^), or magnetic resonance imaging (MRI) brain features of encephalitis with either hyperintense signal on T2-weighted fluid-attenuated inversion recovery sequences highly restricted to 1 or both medial temporal lobes (limbic encephalitis) or in multifocal areas involving gray matter, white matter, or both compatible with demyelination or inflammation.

Failure to fulfill both part 1 and 2 of the criteria precludes a diagnosis of any category of autoimmune encephalitis. Part 3 of the autoimmune encephalitis diagnostic criteria was not analyzed as this component specifies reasonable exclusion of alternative diagnoses, which by design of the present study would be difficult to quantify retrospectively.

Data collected included age at symptom onset, sex, and time from disease onset to correct diagnosis, insidious (symptoms developing over ≥3 months) vs subacute (<3 months) onset, cancer history, thyroid autoimmunity, or other autoimmune disorders. Results of neuropsychological testing were classified as normal (for age and education) or abnormal. We collected data on elevated IgG index, CSF-restricted oligoclonal bands, electroencephalogram (categorized as normal, showing epileptiform activity [clinical or subclinical seizures, spikes, or sharp waves], slowing or other findings), thyroid peroxidase antibodies, other serologic evidence of systemic autoimmunity, and serum and CSF anti-neural or glial antibodies (including information on titer and assay type when available). Brain biopsy or autopsy details were obtained when applicable. Information on immunotherapies and adverse reactions were also collected.

Participating sites selected from the following potential reasons for misdiagnosis in each patient: (1) overinterpretation of a nonspecific positive antibody; (2) failure to accept an alternative psychiatric diagnosis; (3) misclassification of functional neurologic symptoms as true neurologic abnormalities; (4) overinterpretation of nonspecific cognitive symptoms as encephalitis; or (5) other. There was also a free text section for additional reasons for misdiagnosis.

### Statistical Analysis

Descriptive statistics were used. For categorical variables, frequency and percent were used, whereas for continuous variables, median and range or interquartile range were used. JMP Pro, version 14.1.0 (JMP Statistical Discovery LLC) was used.

## Results

### Demographics and Clinical Characteristics

We included 107 patients misdiagnosed as having autoimmune encephalitis at the 6 participating centers. The median (IQR) age at symptom onset was 48 (35.5-60.5) years and 65 (61%) were female. The median (IQR) time from onset to the correct diagnosis was 16 (7-40) months. A history of any type of autoimmune disease was noted in 44 individuals (41%), of whom 34 (77%) had thyroid autoimmunity. Six patients (6%) had a history of cancer outside of the nervous system. Symptom onset was insidious in 51 of 107 patients (48%), although some had superimposed subacute worsening.

### Frequency of Misdiagnosis Compared With Confirmed Diagnoses of Autoimmune Encephalitis 

Autoimmune encephalitis misdiagnosis occurred in 107 individuals during a period over which 286 were correctly diagnosed as having autoimmune encephalitis. This included Mayo Clinic (misdiagnosis, 44; true diagnosis, 100); University of Oxford (misdiagnosis, 18; true diagnosis, 125); University of Texas Southwestern (misdiagnosis, 18; true diagnosis, 19); University of California, San Francisco (misdiagnosis, 17; true diagnosis, not available); Washington University in St Louis (misdiagnosis, 6; true diagnosis, 42); and University of Utah (misdiagnosis, 4; true diagnosis, not available).

### Disorders Misdiagnosed as Autoimmune Encephalitis 

Alternative diagnoses are detailed in Table 1, with imaging examples in the Figure. Of 107 patients, 17 (16%) had a definite alternative diagnosis confirmed on biopsy (astrocytoma, 6; lymphoma, 2; medulloblastoma, 1; neuronal intranuclear inclusion disease, 1), autopsy (Creutzfeldt-Jakob disease, 1; Alzheimer disease, 1), with genetic testing (mitochondrial encephalomyopathy lactic acidosis and strokelike episodes, 2; behavioral variant frontotemporal dementia with genetic confirmation of a valosin containing protein variant, 1), infectious testing (HIV positive, 1) and other laboratory testing (thiamine deficiency, 1). The remaining 90 alternative clinical diagnoses were often supported by laboratory testing and imaging and are demonstrated by the cases highlighted in Figure E and F.

**Table 1.** Alternative Final Diagnoses in Those Initially Misdiagnosed as Autoimmune Encephalitis

Alternative diagnosis	No. (%)	
	Individuals with initial diagnosis (n = 107)	Individuals who fulfilled possible autoimmune encephalitis criteria (n = 30)
Functional neurologic disorder	27 (25)	6 (22)
Neurodegenerative dementia	22 (20.5)	5 (23)
Alzheimer disease^a^	6	0
Dementia with Lewy bodies^b^	4	1
Behavioral variant frontotemporal dementia	4	2
Creutzfeldt-Jakob disease	2	1
Vascular cognitive impairment	1	0
Other^c^	5	1^c^
Psychiatric disease	19 (18)	2 (11)
Depression^d^	7	2
Anxiety	3	0
Schizophrenia	2	0
Bipolar	2	0
Other^e^	5	0
Nonspecific cognitive syndrome in the setting of ≥1 of fibromyalgia, chronic fatigue, sleep disorder, medication adverse reaction, or other comorbidity^f^	11 (10)	1 (9)^f^
Neoplasm	10 (9.5)	7 (70)
Glioma (glioblastoma, astrocytoma, or not otherwise specified)^g^	7	5
Primary central nervous system lymphoma	2	2
Cerebellar medulloblastoma with cerebellar cognitive syndrome	1	0
Seizure disorder, nonimmune-mediated^h^	5 (4.5)	3 (60)
Infectious	3 (2.5)	1 (33)
Residua of prior viral encephalitis	2	1
HIV leukoencephalopathy	1	0
Mitochondrial encephalomyopathy lactic acidosis and strokelike episodes	2 (2)	1 (50)
Other metabolic	2 (2)	1 (50)
Adrenal insufficiency	1	0
Wernicke encephalopathy	1	1
Other	6 (6)	3 (50)
Small vessel vasculitis	2	0
Klein Levin syndrome	1	0
Nonimmunotherapy responsive progressive cerebellar degeneration with cerebellar cognitive syndrome	1	1
Multiple sclerosis and depression	1	1
Nonimmune encephalopathy without further classification	1	1

^a^ One individual had coexisting vascular cognitive impairment; 1 patient with prior typical anti-LGI1 encephalitis developed an insidious dementia in follow-up that was suspected to be recurrent autoimmune encephalitis, but repeat LGI1 antibodies testing results were negative (and thus we categorized as antibody negative for this study), and the patient did not respond to immunotherapy and autopsy later confirmed Alzheimer disease as the cause of the insidious dementia.

^b^ Two individuals were suspected to have comorbid Alzheimer disease.

^c^ Progressive supranuclear palsy, 1; neuronal intranuclear inclusion disease, 1 (this patient fulfilled criteria for possible autoimmune encephalitis); primary lateral sclerosis with cognitive impairment, 1; amnestic mild cognitive impairment, 1; neurodegenerative unclassifiable, 1.

^d^ Two individuals had psychosis, one of which also had catatonia.

^e^ Depression and anxiety in combination, 1; developmental delay with regression, 1; psychiatric disease without classification, 3.

^f^ Other contributors included migraine headaches, insomnia, and psychiatric comorbidity; in this category, there were often multiple combinations of these factors contributing.

^g^ In 1 patient, biopsy confirmation was not available.

^h^ One from multiple cavernous malformations.

**Figure.**
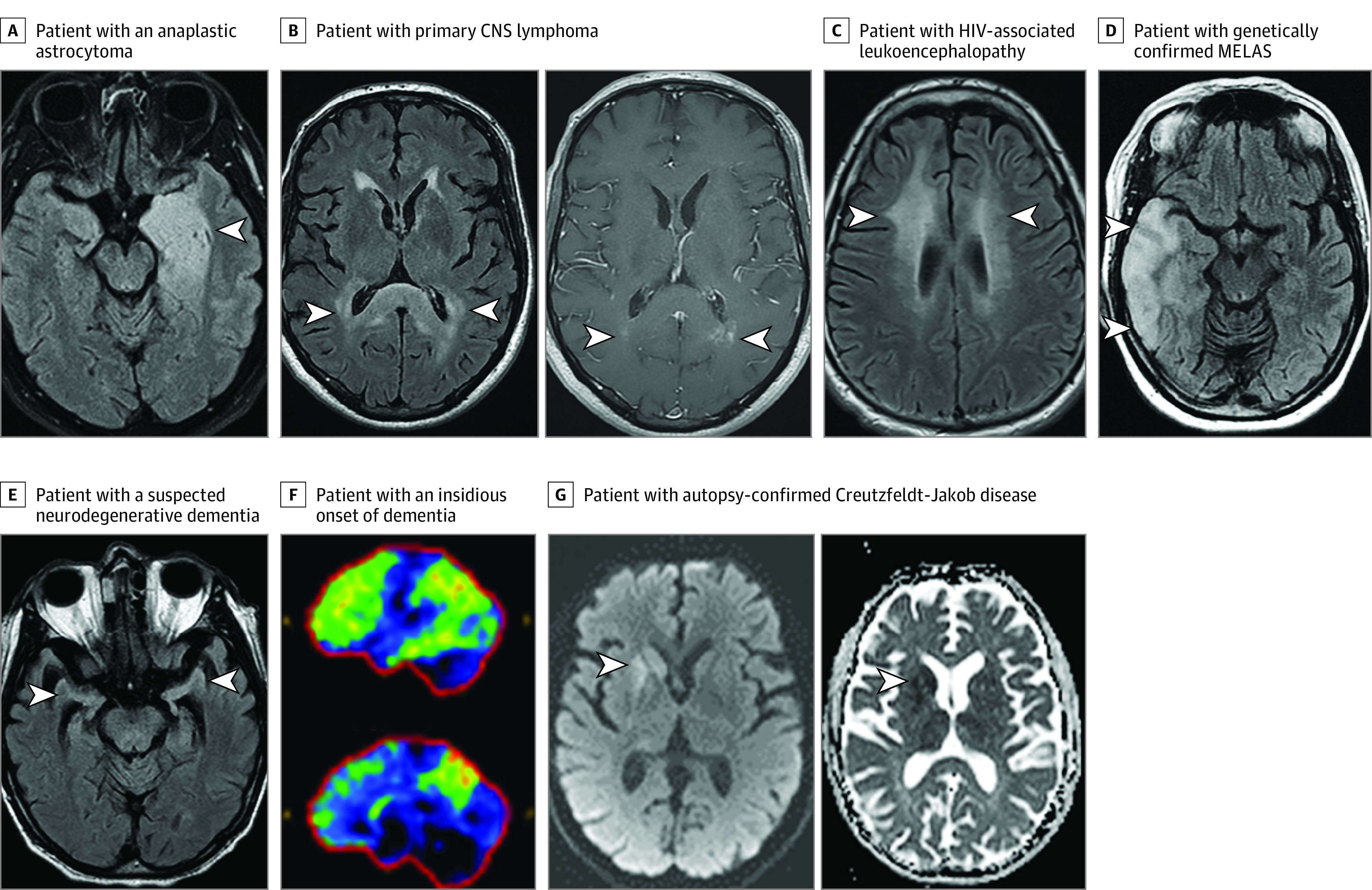
Imaging Examples of Patients Who Were Initially Thought to Have Autoimmune Encephalitis but Later Had an Alternative Diagnosis Made A T2-weighted axial fluid-attenuated inversion recovery (T2-FLAIR) image reveals a left mesial temporal lobe T2-hyperintensity and swelling (A, arrowhead) in a patient with an anaplastic astrocytoma. Note in retrospect the fullness/enlargement of the affected region, possibly suggesting some mass effect. Axial T2-FLAIR image reveals bilateral splenium T2-hyperintensity (B, left panel, arrowheads) with multifocal punctate enhancement (B, right panel, arrowheads) in a patient with primary central nervous system (CNS) lymphoma. An axial T2-FLAIR image reveals bilateral confluent T2-hyperintensity in the subcortical white matter (C, arrowheads) in a patient with HIV-associated leukoencephalopathy. Axial T2-FLAIR image reveals right temporal cortical swelling and T2-hyperintensity (D, arrowheads) in a patient with genetically confirmed mitochondrial encephalomyopathy, lactic acidosis, and stroke-like episodes (MELAS). An axial T2-FLAIR image shows disproportionate bilateral hippocampal atrophy (E, arrowheads) in a patient with a suspected neurodegenerative dementia with features potentially consistent with mixed Alzheimer disease and dementia with Lewy bodies. ^18^F-Fluorodeoxyglucose positron emission tomography reveals reduced uptake of glucose (normal, dark blue/black; mildly reduced, green; moderately reduced, yellow; severely reduced, red) in the frontotemporoparietal region, precuneus and posterior cingulate (F) most suspicious for underlying Alzheimer disease in a patient with an insidious onset of dementia and elevated cerebrospinal fluid phospho-Tau and low cerebrospinal fluid amyloid-β 42 also suggestive of this diagnosis. Axial diffusion weighted hyperintensity (G, left panel) and apparent diffusion coefficient hypointensity (G, right panel) consistent with restricted diffusion in the right caudate and putamen in a patient in whom autopsy later confirmed Creutzfeldt-Jakob disease.

### Fulfillment of Diagnostic Criteria for Possible Autoimmune Encephalitis 

Those fulfilling part 1 of the criteria had 1 or more of a clinical presentation of a subacute onset (rapid progression of <3 months) with 1 or more of working memory deficits (short-term memory loss) (36 [34%]), altered mental status (43 [40%]), or psychiatric symptoms (42 [39%]).

Those fulfilling part 2 of the criteria had 1 or more of the following: (1) focal central nervous system findings in 31 patients (29%); (2) seizures not explained by a previously known seizure disorder in 26 patients (24%); (3) CSF pleocytosis in 16 of 84 patients (19%); or (4) MRI brain features suggestive of encephalitis in 19 of 104 patients (18%) with either features of limbic encephalitis in 10 (Figure A) or multifocal abnormalities compatible with demyelination or inflammation in 9 (Figure B-D).

In total, 77 patients (72%) did not fulfill autoimmune encephalitis diagnostic criteria as they lacked requirements for possible autoimmune encephalitis diagnosis, which is a prerequisite for any other autoimmune encephalitis diagnostic category.

### Antibody Testing

Thyroid peroxidase antibodies were positive in 24 of 62 individuals (39%). Nineteen patients had coexisting serologic evidence of systemic autoimmunity with antinuclear antibody positivity most common. Neural autoantibodies were identified more often in serum (48 of 105 [46%]) than CSF (7 of 91 [8%]) and are outlined in Table 2.

**Table 2.** Positive Neural Antibodies That Contributed to Misdiagnosis of Autoimmune Encephalitis

Positive neural antibody	No.^a^	Assay detection method	Quantitative results with median (range)^b^	Reference range^b^
Serum				
GAD65	14	RIA	0.10 (0.07-45.6) nmol/L^c^	≤0.02 nmol/L
Voltage-gated potassium-channel-complex (negative for LGI1 & CASPR2)	10	RIA	0.11 (0.07-1.03) nmol/L^c^	≤0.02 nmol/L
NMDAR^d^	10	CBA	High titer in 4; moderate titer in 1; low titer in 1; unavailable titer in 4	Negative
Ganglionic acetylcholine receptor	5	RIA	0.1 (0.05-0.12) nmol/L^e^	≤0.02 nmol/L
CASPR2^f^	2	CBA	Low titer in both	Negative
LGI1^f^	2	CBA	Low titer in both	Negative
Muscle acetylcholine receptor	2	RIA	0.27 and 0.44 nmol/L	≤0.02 nmol/L
Voltage-gated calcium channel (N type)	2	RIA	0.16 and 0.27 nmol/L	≤0.03 nmol/L
Striated muscle	2	ELISA	1:480	<1:240
Glycine receptor	1	CBA	NA	Negative
Amphiphysin^d^	1	WB	NA	Negative
Multiple positive neural antibodies in noncertified laboratory	1	Uncertain	NA	Negative
CSF				
NMDAR^d^	4	CBA	Low titer in 1; unavailable titer in 3	Negative
Voltage-gated potassium-channel-complex (Negative for LGI1, CASPR2)	1	RIA	Not available	≤0.02 nmol/L
GAD65	1	RIA	3.01 nmol/L	≤0.02 nmol/L
Unclassified neural antibody	1	TIFA	Not available	Negative

Abbreviations: CASPR2, contactin-associated protein-like 2; CBA, cell-based assay; CSF, cerebrospinal fluid; ELISA, enzyme-linked immunosorbent assay; GAD65, glutamic acid decarboxylase 65; LGI1, leucine-rich-glioma-inactivated-1; NA, not applicable; NMDAR, *N*-methyl-d-aspartate receptor; RIA, radioimmunoprecipitation assay; TIFA, tissue-based immunofluorescence assay; WB, western blot.

^a^ As the exact details of what antibodies were tested in each patient were not always available, no denominator or percentage is given here and some patients had more than 1 antibody detected.

^b^ For antibodies detected by RIA and ELISA, only values and reference ranges from the Mayo Clinic neuroimmunology laboratory were used; for CBA, the standard reference range of negative is similar across all laboratories, although for the quantitative result, some report a binary result of positive or negative and others quantify by low, moderate, or high positive, which were provided when available.

^c^ Available in 5 individuals.

^d^ Not evident on mouse tissue-based immunofluorescence assay.

^e^ Available in 3 individuals.

^f^ Both patients had final diagnoses of functional neurologic disorder.

### Additional Investigations

Neuropsychological test results were abnormal in 38 of 54 patients (70%). Electroencephalogram findings were abnormal in 31 of 79 (39%) and revealed epileptiform abnormalities in 16 and slowing in 9; details of abnormalities were not available in 6 patients. CSF-restricted oligoclonal bandings or IgG index positivity occurred in 7 of 82 (9%) tested.

### Additional Clinical Details on Patients With a CSF Antibody

The 4 patients with *N*-methyl-d-aspartate receptor (NMDAR) antibodies in the CSF without evidence on mouse tissue-based indirect immunofluorescence had HIV-associated leukoencephalopathy (Figure C), pathologically confirmed anaplastic astrocytoma, functional neurologic disorder, and behavioral variant frontotemporal dementia, respectively. In all 4 patients, NMDAR antibodies were also detected in serum. One patient with an unclassified CSF antibody on immunohistochemistry had a progressively enlarging brain mass without immunotherapy response with imaging consistent with glioma (final pathology was not available). One patient with CSF GAD65 antibodies (titer, 3.01 nmol/L; normal, ≤0.02 nmol/L) had mixed vascular cognitive impairment and symptomatic Alzheimer disease (CSF biomarker confirmed). Finally, 1 patient with VGKC autoantibodies (LGI1 and CASPR2 negative) had cryptogenic epilepsy (not immune-related).

### Treatment Details

One or more immunotherapies were used in 84 of 107 patients (79%) with treatment-related adverse reactions documented in 17 of 84 patients (20%) (Table 3).

**Table 3.** Treatments Used for Autoimmune Encephalitis and Associated Adverse Reactions

Type of treatment used	No. of patients who received ≥1 of each treatment (n = 84)	Types and frequency of documented adverse reactions^a^
Corticosteroids (intravenous, oral, or both)	78	Steroid-related psychosis or agitation, 5; mania, 1; depression, 1; gastritis, 1; avascular necrosis of the hip, 1; insomnia, 1; heart failure, 1; colonic fistula, 1; myopathy, 1
Intravenous immunoglobulin	30	Aseptic meningitis, 2; alopecia, 1; confusion, 1
Plasma exchange	16	NA
Mycophenolate mofetil	11	NA
Rituximab	10	Headache, 1
Azathioprine	2	Nausea, 1
Cyclophosphamide	2	NA
Methotrexate	1	NA
Adrenocorticotropic hormone	1	NA

Abbreviation: NA, not applicable.

^a^ Given the details were obtained from medical record review at the time of misdiagnosis, this could underestimate the number of adverse reactions.

### Reasons for Misdiagnosis

The reasons for misdiagnosis included 1 or more of overinterpretation of a nonspecific positive antibody result (53 [50%]); misinterpretation of nonspecific symptoms as neurologic (19 [18%]); imaging findings felt to be consistent with autoimmune encephalitis (15 [14%]); functional neurologic features mistaken for true neurologic symptoms (14 [13%]); abnormal cerebrospinal fluid findings (9 [8%]); psychiatric manifestations thought to be from autoimmune encephalitis (8 [7%]); failure to accept a psychiatric diagnosis (5 [5%]); or subacute onset or fluctuating course (4 [4%]).

## Discussion

This study highlights that misdiagnosis of autoimmune encephalitis is an important and frequent clinical problem. Autoimmune encephalitis misdiagnosis was identified at participating subspecialty outpatient clinics, but the initial incorrect autoimmune encephalitis diagnosis occurred at both outside facilities and participating centers. This shows that misdiagnosis of autoimmune encephalitis can be encountered in multiple settings, including at autoimmune neurology subspeciality clinics with focused expertise. Many of these patients endured a delay to their correct diagnosis for longer than a year, and one-fifth experienced morbidity related to unnecessary immunotherapy. Overinterpretation of a nonspecific autoantibody was a frequent contributor to misdiagnosis. In 72% of patients, they did not fulfill autoimmune encephalitis diagnostic criteria, suggesting more stringent adherence to these criteria may prevent misdiagnoses. In particular, an insidious onset of symptoms and absence of MRI or CSF findings suggestive of neuroinflammation should raise suspicion for an alternative diagnosis. Yet, patients with LGI1 (the most common form of autoimmune encephalitis), CASPR2m and IgLON5 antibodies can present over long durations with minimal evidence of paraclinical investigation abnormalities, other than the autoantibody itself.^11,12,13,14^

Autoimmune encephalitis is a rare condition, with a cumulative incidence of approximately 3 to 9 per million person-years and common conditions accounted for a high proportion of cases mistaken for autoimmune encephalitis.^2,15,16^ This is similar to recent data concerning multiple sclerosis misdiagnosis.^17^ Functional neurologic disorders and psychiatric diseases are highly prevalent alternative diagnoses whose distinction from autoimmune encephalitis can be challenging.^18,19,20,21^ Autoimmune encephalitis is increasingly considered in patients with psychiatric symptoms as it is potentially treatable with immunotherapy, but autoimmune encephalitis is much less common than primary psychiatric disease, for instance, accounting for less than 1% presenting with a typical first episode of psychosis.^22,23^ Psychiatric disease combined with other contributors to cognitive deficits such as chronic pain, sleep disturbance, and medication adverse reactions also led to misdiagnosis. Such patients often had normal neuropsychological testing and did not fulfill autoimmune encephalitis diagnostic criteria due to absence of MRI and CSF findings suggesting classic neuroinflammation.

Neurodegenerative disorders accounted for 20% of misdiagnoses and the insidious onset and absence of neuroinflammation on testing help discriminate from autoimmune encephalitis. However, fluctuations in patients with Lewy body disease and rapid progression with overlapping MRI findings in Creutzfeldt-Jakob disease can make this distinction challenging.^24^ Imaging and CSF analysis for amyloid and tau and CSF prion detection with real-time quaking-induced conversion are novel biomarkers that aid diagnosis of Alzheimer disease and Creutzfeldt-Jakob disease, respectively.^25,26^

We found 28% of patients fulfilled autoimmune encephalitis criteria and such patients usually had overlapping MRI or CSF findings with autoimmune encephalitis. Temporal lobe glioma may mimic autoimmune encephalitis; however, the absence of sustained response to immunotherapy, presence of mass effect on MRI (Figure, A) and lack of CSF inflammation may inform the correct diagnosis.^8^ The multifocal MRI abnormalities, CSF pleocytosis, and steroid responsiveness of central nervous system lymphoma mimicked autoimmune encephalitis here and previously.^27^ The subacute encephalopathy, cortical swelling, and signal abnormality on MRI with mitochondrial encephalomyopathy lactic acidosis and strokelike episodes mimicked autoimmune encephalitis similar to prior reports.^28^ Seizure-related MRI signal abnormalities can overlap with autoimmune encephalitis MRI findings and lead to misdiagnosis.^29^ Thiamine deficiency and HIV infection are important treatable mimics identified here and reported previously.^30,31^ Taken together, the aforementioned cases pose a particular challenge given the paraclinical features in common with autoimmune encephalitis.

Overinterpretation of a nonspecific antibody was the largest potential contributor to autoimmune encephalitis misdiagnosis and a list of the more problematic antibodies are summarized in the Box. Thyroid peroxidase antibodies occur in 13% of people and 20% older than 60 years, which drastically diminishes their diagnostic utility in autoimmune encephalitis or Hashimoto encephalopathy and positive results often contribute to misdiagnosis.^5,32^ With neural autoantibody biomarkers the diagnostic accuracy varies by pretest probability, sample assessed (serum or CSF), antibody type, assay methodology, and antibody titer.^6^ As up to 5% of patients may harbor a positive neuronal antibody, clinically irrelevant results may be frequent if many patients are serologically assessed.^6,33^ Indeed, in this study, some positives (eg, ganglionic acetylcholine receptor antibodies) were misinterpreted as being relevant despite autoimmune encephalitis not being the typical phenotype, suggesting that removing problematic antibodies with low specificity from autoimmune encephalitis autoantibody panels could reduce misdiagnosis.^34,35,36,37^ Low-end titer serum GAD65 antibody positives were often overinterpreted as supporting autoimmune encephalitis but occur in 8% of the population (particularly individuals with diabetes) and typically only high titer (>10 000 IU/mL using enzyme-linked immunosorbent assay or >20 nmol/L using radioimmunoassay)^38,39^ serum positives or CSF detection are neurologically relevant.^40,41,42^ Laboratories offering serum GAD65 antibody testing for neurologic indications should consider using these higher cutoffs for neurologically relevant positivity. Voltage-gated potassium channel complex antibody positivity without LGI1 or CASPR2 reactivity are not useful for autoimmune encephalitis diagnosis,^43,44^ while low-titer CASPR2 antibodies are also problematic and only high titers support autoimmune encephalitis.^45,46,47^ Serum NMDAR antibodies with negative CSF results were a red flag here, as noted previously.^48^ Rarely, CSF NMDAR antibodies by cell-based assay alone led to misdiagnosis. Despite its high specificity, these positive results in CSF may relate to diffusion of high serum levels, rather than intrathecal synthesis. Detection using a second rodent tissue-based assay enhances CSF NMDAR antibody specificity further.^48^ Antibodies detected by western blot/line blot or immunoblot in isolation often yield false positives and require cautious interpretation.^49,50^ Moreover, detection of neural antibodies in noncertified laboratories require extreme caution. While this study focused only on autoimmune encephalitis, overinterpretation of nonspecific antibodies is also problematic in other neurologic syndromes in which antibodies are tested (eg, ataxia, myelopathy, stiff person syndrome, peripheral nervous system disorders). Increased education of neurologists on when to order neural autoantibodies and how to interpret positive results is needed to reduce the risk of misdiagnosis and interpretative comments provided by laboratories reporting results can be helpful in this regard.^4,51,52^

Box. **Summary of Red Flags in Autoimmune Encephalitis Diagnosis**

**Clinical**

- Insidious onset
- Multiple comorbidities that cause cognitive deficits such as polypharmacy, chronic pain, fibromyalgia, sleep disorders
- Examination results consistent with functional neurologic disorder
- Features of mitochondrial disease present
- Normal neuropsychological test results

**Magnetic Resonance Imaging of the Head**

- Normal
- Progressive atrophy without signal abnormalities or enhancement
- Lesion(s) continuing to expand despite immunotherapy

**Cerebrospinal Fluid**

- Noninflammatory^a^

**Serology**

- TPO antibodies of any titer
- Low titer–positive GAD65 antibodies
- Voltage-gated potassium channel complex antibodies negative for LGI1/CASPR2
- Low-titer antibody positives by older generation techniques (eg, RIA)
- Isolated serum NMDAR antibody negative in CSF
- Immunoblot or line blot antibody positivity in isolation
- Low titer positive–CASPR2 antibodies
- Antibody detection in noncertified laboratories

Abbreviations: CASPR2, contactin-associated protein-like 2; CSF, cerebrospinal fluid; GAD65, glutamic acid decarboxylase 65; LGI1, leucine-rich-glioma-inactivated-1; NMDAR, *N*-methyl-d-aspartate receptor; RIA, radioimmunoprecipitation assay; TPO, thyroid peroxidase.

^a^
Normal white blood cell count and absence of CSF unique oligoclonal bands.

Autoimmune encephalitis misdiagnosis is problematic for multiple reasons. First, misdiagnosis of autoimmune encephalitis increases morbidity from failure to treat the actual diagnosis. Second, immunosuppressant treatments commonly have adverse reactions that may be serious, and in this study included infection, psychosis, avascular necrosis of the hip, and heart failure. Moreover, there are many less severe, yet common and bothersome, adverse reactions of corticosteroids including insomnia, weight gain and irritability, some of which may not have been captured in this analysis. Third, during the COVID-19 pandemic, immunotherapies may increase risk of severe COVID-19 infection and hinder vaccine and natural infection responses.^53,54^ Finally, increased health care costs may arise from the use of expensive immunosuppressants or unnecessary evaluation for an underlying cancer prompted by nonspecific antibody detection.

### Limitations

The retrospective design was a limitation and prospective studies are needed to assess autoimmune encephalitis misdiagnosis frequency and characteristics among new referrals to subspecialty clinics with presumed autoimmune encephalitis. Such studies could incorporate probable and definite categories of autoimmune encephalitis diagnostic criteria to better discriminate true autoimmune encephalitis from autoimmune encephalitis misdiagnosis.^1^ The selection bias of analyzing autoimmune encephalitis misdiagnosis identified at subspecialty autoimmune neurology clinics could underestimate the rate of autoimmune encephalitis misdiagnosis and it may exceed true autoimmune encephalitis diagnosis in the general population. There are many potential contributors to underrepresentation of autoimmune encephalitis misdiagnosis including our requirement for an in-person visit as autoimmune encephalitis misdiagnosis can be identified in other settings (eg, video visit, electronic medical record review, other communication between physicians). Moreover, during triage for appointments, true autoimmune encephalitis may be favored over cases suspected to be misdiagnosed. Also, infectious mimics of autoimmune encephalitis are more likely to be encountered in hospitalized patients and our study focused on those identified at outpatient clinics.^55^ Finally, differences in rates of autoimmune encephalitis misdiagnosis across centers likely reflect variation in referral patterns. Further studies are needed to better capture autoimmune encephalitis misdiagnosis rates across other settings.

## Conclusions

In summary, neurologists should be aware of the potential for autoimmune encephalitis misdiagnosis and consider a broad differential diagnosis including common disorders when evaluating suspected cases. Improved recognition of the clinical, imaging, and serologic red flags in the evaluation of autoimmune encephalitis summarized in the Box may lessen the burden of misdiagnosis in the future.
